# An analysis of early developmental trauma in social anxiety disorder and posttraumatic stress disorder

**DOI:** 10.1186/1744-859X-13-16

**Published:** 2014-05-29

**Authors:** Melanie Bishop, David Rosenstein, Susanne Bakelaar, Soraya Seedat

**Affiliations:** 1 Department of Psychiatry, Faculty of Medicine and Health Sciences, Tygerberg Campus, Stellenbosch University, Cape Town 7505, South Africa

**Keywords:** Childhood trauma, Social anxiety, PTSD, Early developmental trauma, Anxiety

## Abstract

**Background:**

The early contributions of childhood trauma (emotional, physical, sexual, and general) have been hypothesized to play a significant role in the development of anxiety disorders, such as posttraumatic stress disorder (PTSD) and social anxiety disorder (SAD). The aim of this study was to assess childhood trauma differences between PTSD and SAD patients and healthy controls, as measured by the Early Trauma Inventory.

**Methods:**

We examined individuals (*N* = 109) with SAD with moderate/severe early developmental trauma (EDT) (*n* = 32), individuals with SAD with low/no EDT (*n* = 29), individuals with PTSD with EDT (*n* = 17), and healthy controls (*n* = 31). The mean age was 34 years (SD = 11). Subjects were screened with the Mini-International Neuropsychiatric Interview (MINI), Liebowitz Social Anxiety Scale (LSAS), Clinician-Administered PTSD Scale (CAPS), and Childhood Trauma Questionnaire (CTQ). Analysis of variance was performed to assess group differences. Correlations were calculated between childhood traumas.

**Results:**

Although not statistically significant, individuals with PTSD endorsed more physical and sexual childhood trauma compared with individuals with SAD with moderate/severe EDT who endorsed more emotional trauma. For all groups, physical and emotional abuse occurred between ages 6 and 11, while the occurrence of sexual abuse in individuals with PTSD was at 6–11 years and later (13–18 years) in individuals with SAD with moderate/severe EDT. For emotional abuse in all groups, the perpetrator was mostly a primary female caregiver; for sexual abuse, it was mostly a nonfamilial adult male, while for physical abuse, it was mostly a caregiver (male in PTSD and female in SAD with moderate/severe EDT).

**Conclusions:**

The contribution of childhood abuse to the development of PTSD and SAD and the differences between these groups and other anxiety disorders should not be ignored and attention should be given to the frequency and severity of these events. The relationship of the perpetrator(s) and the age of onset of childhood abuse are also important considerations as they provide a useful starting point to assess impact over the life course. This can, in turn, guide clinicians on the optimal timing for the delivery of interventions for the prevention of PTSD and SAD.

## Introduction and background

Early developmental trauma (EDT) or childhood trauma may loosely be defined as any traumatic experience that occurs before 18 years of age [1]. EDT has been linked to the development of anxiety disorders in adulthood [2-5]. Among South Africans, anxiety disorders (15.8%) are the most prevalent lifetime disorders according to the South African Stress and Health (SASH) study, with social anxiety disorder (SAD) at 2.8% and posttraumatic stress disorder (PTSD) at 2.3% [6]. It has been estimated that one in ten children, on average, is neglected or psychologically abused annually and that approximately 4% to 16% are physically abused [7]. Stein et al. in 1996 found in their sample that both adult males and females with an anxiety disorder had higher rates of childhood physical abuse than those without an anxiety disorder [8]. In addition, females with anxiety disorders had higher rates of childhood sexual abuse [8]. Also, Prigerson et al. in 1996 found that psychological abuse and parental loss were risk factors for the development of adult psychiatric disorders [5]. In SAD [9] and PTSD [10], childhood traumas include physical abuse [11-13], sexual abuse [12-18], and emotional abuse [19-23].

### Physical and sexual abuse in PTSD and SAD

The association between different subtypes of EDT and anxiety disorders in adults is a complex one. For example, in a national study by Cougle et al. in 2010, childhood physical abuse was specifically associated with PTSD in adults [11]. Furthermore, there was a relationship between sexual and physical abuse experienced in childhood and adult PTSD [11]. Sexual abuse in childhood has also been established to be a risk factor for the development of SAD [18] and PTSD [12,13,15,16] in adulthood. In a study of women from an American cohort, childhood sexual abuse occurred in approximately 10% of women diagnosed with PTSD [17]. This was supported by the findings of Cutajar et al. in 2010 who found sexual abuse was most strongly correlated with PTSD [16]. Also, significantly more females than males who experienced childhood sexual abuse were diagnosed with PTSD [16]. In a separate study, adults with SAD reported higher rates of sexual abuse in childhood compared to healthy controls [14].

### Emotional and other abuse in PTSD and SAD

Childhood emotional abuse has been reported to correlate more strongly, than either physical or sexual abuse, with a diagnosis of SAD and depression [20]. According to Kuo et al. in 2011 individuals with SAD had higher rates of childhood emotional abuse and emotional neglect compared to healthy controls [21]. In one study conducted by Simon et al. in 2009, 56% and 39% of individuals with SAD, respectively, experienced childhood emotional abuse and neglect [22]. Kuo et al. in 2011 investigated the frequency of childhood traumas (sexual abuse, physical abuse, physical neglect, emotional abuse, and emotional neglect) as measured by the Childhood Trauma Questionnaire (CTQ) [21]. As mentioned previously, they found that childhood emotional abuse and neglect were more frequently reported by individuals with SAD [21]. Other childhood traumas have been reported in PTSD and SAD. Afifi et al. in 2009 found that a combination of childhood abuse and parental divorce significantly increased the likelihood of a diagnosis of lifetime PTSD in adulthood [24]. Binelli et al. in 2012 found only one significant positive correlation between family violence and SAD, although the assessment of childhood trauma was based on five closed questions (yes/no) relating to five childhood adversities, namely, the loss of someone close, emotional abuse, physical abuse, family violence, and sexual abuse [25].

### Age of onset and relationship to perpetrator in PTSD and SAD

The association between EDT subtypes, age of occurrence, and relationship to the perpetrator has not received much attention in the literature [26]. In individuals with PTSD, the onset of childhood sexual abuse (CSA) seems to be between the ages of 6 and 13 years, with a mean duration of 7 years [27]. In one study the age of onset of CSA was approximately 7 years [26]. Also, most perpetrators (87%) of CSA in adults with PTSD were males and were nuclear family members (37%), followed by nonfamily members (35%), and extended family members (28%) [27]. In another study, the age of onset of abuse by a family friend/other perpetrators was significantly associated with social anxiety in adulthood [28]. Most were abused by other perpetrators (e.g., acquaintances, boyfriends, and babysitters) (44%), followed by strangers (40%), other relatives or family members (29%), family friends (16%), and least by their fathers (14%). Furthermore, the average age of onset was 9.85 years [28].

### Rationale and aims

Although EDT and other early childhood adversities have been examined in SAD and PTSD, there have been no comparative studies of the frequency and association of EDT subtypes in these disorders. The clinical value of such a comparison between childhood trauma in PTSD and SAD is important as it provides clinicians with information that can be used in interventions with patients with PTSD and SAD. The aim of this study was to assess EDT differences between PTSD and SAD patients and healthy controls. Secondly, we wanted to establish the frequency of events occurring in childhood, as measured by the Early Trauma Inventory (ETI). Thirdly, we wanted to establish the age of onset of childhood abuse and type of perpetrator (relationship to victim and gender) in this sample with SAD and PTSD. Lastly, we correlated childhood traumas as measured by the ETI and childhood traumas measured by the CTQ. We hypothesized that there would be significant group differences, with more childhood trauma overall, and significantly more sexual trauma, reported by the PTSD group. We further hypothesized that (i) the age of onset of EDT and the perpetrators involved would differ between the groups and (ii) childhood trauma as measured by the CTQ would be significantly correlated on the ETI.

## Method

### Design

This study used clinical data obtained from a larger imaging genetics project whose primary aim was to evaluate group differences in functional magnetic imaging responses in the amygdala by genotype.

### Participants

Participants were recruited from various community clinics, psychiatric institutions, hospitals, and NGOs. Participants were also recruited through advertising (print, electronic, and media). Participants were recruited over a 3-year period (2010–2013). A total of 109 participants (31 healthy controls, 32 with SAD and moderate/severe EDT, 29 with SAD with low/no EDT, and 17 with PTSD resulting from EDT) were selected in the basis of the presence or absence of SAD/PTSD and the presence/absence of early childhood trauma, using the Mini-International Neuropsychiatric Interview (MINI), the Liebowitz Social Anxiety Scale (LSAS), the Clinician-Administered PTSD Scale (CAPS), and the CTQ. Healthy controls were selected on the basis of absence of any psychiatric disorder and early childhood trauma, as assessed by the MINI and CTQ (a score below 40 on the CTQ), respectively.

### Assessments

The MINI was administered to assess for SAD and PTSD, and exclude comorbid psychiatric conditions, including substance abuse within the previous 12 months of assessment [29]. The LSAS, with a cut-off score of 60, was used to assess for SAD and distinguish between generalized SAD and specific SAD [30]. This clinician-administered questionnaire assesses current social anxiety disorder through 24 questions on a 4-point Likert scale. The CAPS was used to assess for current and lifetime PTSD [31]. The frequency and intensity of PTSD symptoms (as described by the DSM-IV diagnostic criteria) are measured by CAPS on a separate 5-point Likert scales, each ranging from 0 to 4. These ratings are then summarized into a 9-point severity score for each symptom, ranging from 0–8 [32].

The CTQ and ETI were used to assess EDT and to differentiate the low/no and moderate/severe EDT groups with SAD. A score of less than 40 on the CTQ indicated low/no trauma, a score between 40 and 46 was used as a threshold for exclusion, and a score of more than 46 indicated moderate/severe EDT [1]. The CTQ is a robust screening measure of early childhood trauma. It is a retrospective self-report inventory that assesses the five main areas of early developmental trauma: physical abuse, physical neglect, emotional abuse, emotional neglect, and sexual abuse [1]. The ETI is a detailed clinician-administered structured interview that assesses various aspects of developmental trauma: childhood physical abuse, sexual abuse, emotional abuse, and early major adverse life events such as parental loss or serious illness [33]. The ETI provides a structure to systematically assess specific forms of childhood maltreatment. The advantages of using the ETI over the CTQ are that it is able to assess the age of onset, duration, and severity of each trauma, as well as the relationship of the perpetrator to the victim.

### Ethical considerations

The protocol received ethical clearance from the Health Research Ethics Committee at Stellenbosch University, South Africa. The study was conducted according to internationally and locally accepted ethical guidelines, namely, the Declaration of Helsinki and the South African Department of Health's 2004 Guidelines: *Ethics in Health Research Structures and Processes.* Participation was completely voluntary, and written informed consent was obtained from all participants.

### Statistical analysis

A one-way analysis of variance (ANOVA) was conducted to assess group differences of childhood trauma as measured by the ETI. For significant group differences, Fisher's least significant difference (LSD) *post hoc* testing was administered. Cross-tabulations between groups (control, SAD with low/no EDT, SAD with moderate/severe EDT, and PTSD) within childhood trauma domains as measured by the ETI were done to calculate the frequency of events experienced, age of onset of abuse, and relationship to perpetrators. Furthermore, nonparametric correlations were performed to assess the significance between childhood trauma on the CTQ (emotional abuse, sexual abuse, physical abuse, emotional neglect, physical neglect, and total childhood trauma) and ETI (general abuse, emotional abuse, physical abuse, sexual abuse, and total childhood trauma). All tests were two-tailed for significance, and significance (*p* value) was set at .05.

## Results

### Demographic characteristics

The majority of participants were female (*n* = 60, 55%), of white ethnicity (*n* = 72, 66.1%), and single (*n* = 59, 54.1%). Their age ranged from 20 to 72 years, and the majority had an annual income of more than R60,000 per year (*n* = 76.8%). The average number of years of education was 15 (SD = 3.2), with a range of 5–24 years, and the majority was employed (*n* = 71, 65.1%). All participants were fluent in English. With regards to previously diagnosed psychiatric disorder(s), 64 participants had not previously been diagnosed (57.8%), compared to 45 who were previously diagnosed with a psychiatric disorder (41.3%). One participant did not complete the question (0.9%).

### Abuse characteristics

Significant group differences (PTSD, SAD with moderate/severe EDT, and controls) were found on the ETI in the domains of physical abuse (*p* = 0.00), emotional abuse (*p* = 0.00), sexual abuse (*p* = 0.00), other childhood trauma (*p* = 0.00) and total scores (*p* = 0.00) (Table 1). Fisher's LSD *post hoc* testing was done to establish between-group differences. There were no significant differences between SAD and PTSD groups. Significant differences in each of the trauma domains (physical abuse, emotional abuse, sexual abuse, other childhood trauma, and total trauma) were found between the PTSD and control groups as well as between SAD and control groups (see Table 2). Although not statistically significant, more general, physical, sexual, and total childhood trauma was experienced by the PTSD group. Only emotional abuse was experienced more in the SAD group.

**Table 1:** **Results of the ANOVAs for differences between groups (*N*** **= 80)**

**Variable**	***F *** **value**	***p *** **value**
Childhood physical abuse (ETI physical abuse total)	6.77	0.00**
Childhood emotional abuse (ETI emotional abuse total)	22.11	0.00**
Childhood sexual abuse (ETI sexual abuse)	0.88	0.00**
Total childhood trauma experienced (ETI total score)	1.34	0.00**
Childhood general trauma (ETI general trauma total)	10.6	0.00**

**p < 0.01.

**Table 2:** **LSD *post hoc *** **results of ANOVA between groups (PTSD, SAD with moderate/severe EDT, controls)**

	**Control**^**a**^								**SAD with EDT**^**b**^			
	**SAD with EDT**				**PTSD**				**PTSD**			
	**Significance**	**Mean difference**	**95% CI**		**Significance**	**Mean difference**	**95% CI**		**Significance**	**Mean difference**	**95% CI**	
			**Lower bound**	**Upper bound**			**Lower bound**	**Upper bound**			**Lower bound**	**Upper bound**
Physical abuse (ETI physical abuse total score)	0.00**	-2.003	-3.04	-0.97	0.00**	-2.214	-3.46	-0.97	0.73	–0.211	-1.45	1.02
Emotional abuse (ETI emotional abuse total score)	0.00**	-3.492	-4.46	-2.52	0.00**	-2.918	-4.08	-1.76	0.33	0.574	-0.58	1.73
Sexual abuse (ETI sexual abuse total score)	0.00**	-2.074	-3.37	-0.77	0.00**	-2.767	-4.32	-1.21	0.38	-0.693	-2.24	.85
Total childhood abuse (ETI Total score)	0.00**	-11.016	-15.25	-6.78	0.00**	-13.751	-18.82	-8.68	0.28	-2.735	-7.78	2.31
General trauma (ETI general trauma total score)	0.00**	-4.198	-6.60	-1.80	0.00**	-5.852	-8.73	-2.98	0.25	-1.654	-4.52	1.21

**p < 0.01. ^a^First the control group is compared to the SAD with moderate/severe EDT and PTSD group, respectively; ^b^the SAD with moderate/severe EDT group is compared to the PTSD group.

### Physical abuse

In the total sample, the most prevalent form of physical abuse reported was being spanked with a hand (*n* = 96, 88.1%), followed by being hit or spanked with an object (*n* = 80, 73.4%). This was followed by being burned with a cigarette (*n* = 53, 48.6%) and being pushed or shoved (*n* = 42, 38.5%). This is the same order of frequency reported by the SAD with moderate/severe EDT, SAD low/no EDT, and control groups, respectively. For the PTSD group, the most prevalent childhood physical abuse was being hit or spanked with an object (*n* = 16, 94.1%), followed by being hit with a hand (*n* = 15, 88.2%), and being burned with a cigarette (*n* = 9, 52.9%) or pushed or shoved (*n* = 9, 52.9%) (see Table 3).

**Table 3:** The frequency of events as measured by the ETI

**Item**		**Controls (*n*** **= 31)**	**SAD with no/low EDT (*n*** **= 29)**	**SAD with moderate/severe EDT (*n*** **= 32)**	**PTSD (*n*** **= 17)**	**Total (*N*** **= 109)**
*Physical abuse*						
P1	Spanked with hand	26 (83.9%)	26 (89.7%)	29 (90.6%)	15 (88.2%)	96 (88.1%)
P2	Slapped in face	5 (16.1%)	5 (17.2%)	10 (31.3%)	5 (29.4%)	25 (22.9%)
P3	Burned with cigarette	9 (29.0%)	20 (69%)	15 (46.9%)	9 (52.9%)	53 (48.6%)
P4	Punched or kicked	7 (22.6%)	6 (20.7%)	14 (43.8%)	6 (35.4%)	33 (30.3%)
P5	Hit or spanked with object	18 (58.1%)	21 (72.4%)	25 (78.1%)	16 (94.1%)	80 (73.4%)
P6	Hit with thrown object	3 (9.7%)	3 (10.3%)	10 (31.3%)	6 (35.3%)	22 (20.2%)
P7	Choked	0 (0%)	2 (6.9%)	7 (21.9%)	3 (17.7%)	12 (11%)
P8	Pushed or shoved	9 (29.0%)	9 (31.0%)	15 (46.9%)	9 (52.9%)	42 (38.5%)
P9	Tied up or locked in closet	1 (3.2%)	1 (3.5%)	3 (9.4%)	2 (11.8%)	7 (6.4%)
*Emotional abuse*						
E1	Often put down or ridiculed	7 (22.6%)	11 (37.9%)	26 (81.3%)	11 (64.7%)	55 (50.5%)
E2	Often ignored or made to feel you didn't count	4 (12.9%)	6 (20.7%)	24 (75%)	11 (64.7%)	45 (41.3%)
E3	Often told you are no good	5 (16.1%)	5 (17.2%)	16 (50%)	9 (52.9%)	35 (32.1%)
E4	Often shouted or yelled at	9 (29.0%)	15 (51.7%)	22 (68.8%)	12 (70.6%)	58 (53.2%)
E5	Most of the time treated in cold or uncaring way	2 (6.5%)	2 (6.9%)	19 (59.4%)	9 (52.9%)	32 (29.4%)
E6	Parents control areas of your life	8 (25.8%)	13 (44.8%)	19 (59.4%)	9 (52.9%)	49 (45%)
E7	Parents fail to understand your needs	4 (12.9%)	13 (44.8%)	26 (81.3%)	10 (58.8%)	53 (48.6%)
*Sexual abuse*						
S1	Exposed to inappropriate comments about sex	3 (9.7%)	4 (13.8%)	13 (40.6%)	8 (47.1%)	28 (25.7%)
S2	Exposed to flashing	2 (6.5%)	2 (6.9%)	12 (37.5%)	6 (35.3%)	22 (20.2%)
S3	Spy on you dressing/bathroom	3 (9.7%)	0 (0%)	8 (25%)	2 (11.8%)	13 (11.9%)
S4	Forced to watch sexual acts	0 (0%)	0 (0%)	5 (15.6%)	1 (5.9%)	6 (5.5%)
S5	Touched in intimate parts in way that was uncomfortable	6 (19.4%)	2 (6.9%)	12 (37.5%)	10 (58.8%)	30 (27.5%)
S6	Someone rubbing genitals against you	2 (6.5%)	0 (0%)	8 (25%)	5 (29.4%)	15 (13.8%)
S7	Forced to touch intimate parts	1 (3.2%)	2 (6.9%)	3 (9.7%)	5 (29.4%)	11 (10.1%)
S8	Someone had genital sex against your will	1 (3.2%)	0 (0%)	3 (9.4%)	4 (23.5%)	8 (7.3%)
S9	Forced to perform oral sex	0 (0%)	1 (3.5%)	1 (3.1%)	2 (11.8%)	4 (3.7%)
S10	Someone performed oral sex on you against your will	0 (0%)	0 (0%)	2 (6.5%)	2 (11.8%)	4 (3.7%)
S11	Someone had anal sex with you against your will	0 (0%)	0 (0%)	2 (6.3%)	2 (11.8%)	4 (3.7%)
S12	Someone tried to have sex but didn't do so	1 (3.2%)	0 (0%)	8 (25.8%)	7 (41.2%)	16 (14.7%)
S13	Forced to pose for sexy photographs	1 (3.2%)	0 (0%)	1 (3.1%)	2 (11.8%)	4 (3.7%)
S14	Forced to perform sex acts for money	-	-	-	-	-
S15	Forced to kiss someone in sexual way	0 (0%)	0 (0%)	9 (28.1%)	2 (11.8%)	11 (10.1%)
*General trauma*						
T1	Natural trauma	1 (3.2%)	1 (3.5%)	4 (12.5%)	4 (23.5%)	10 (9.2%)
T2	Serious accidents	6 (19.4%)	6 (20.7%)	7 (21.9%)	7 (41.2%)	26 (23.9%)
T3	Serious personal injury	11 (35.5%)	4 (13.8%)	10 (31.3%)	8 (47.1%)	33 (30.3%)
T4	Serious personal illness	4 (12.9%)	2 (6.9%)	8 (25%)	9 (52.9%)	23 (21.2%)
T5	Death of parent	5 (16.1%)	4 (13.8%)	11 (34.4%)	7 (41.2%)	27 (24.8%)
T6	Serious injury/illness of parent	7 (22.6%)	8 (27.6%)	16 (50%)	10(58.9%)	41 (37.6%)
T7	Separation of parents	3 (9.7%)	5 (17.2%)	13 (40.6%)	6 (35.3%)	27 (24.8%)
T8	Raised in home other than parents	2 (6.5%)	2 (6.9%)	12 (37.5%)	5 (29.4%)	21 (19.3%)
T9	Death of Sibling	2 (6.5%)	4 (13.8%)	7 (21.9%)	3 (17.7%)	16 (14.7%)
T10	Serious illness/injury of sibling	5 (16.1%)	5 (17.2%)	7 (21.9%)	5 (29.4%)	22 (20.2%)
T11	Death of friend	12 (38.7%)	5 (17.2%)	10 (31.3%)	7 (41.2%)	34 (31.2%)
T12	Serious injury of friend	7 (22.6%)	4 (13.8%)	7 (21.9%)	7 (41.2%)	25 (22.9%)
T13	Observe death/serious injury of others	4 (12.9%)	6 (20.7%)	14 (43.8%)	9 (52.9%)	33 (30.3%)
T14	Divorce/separation of parents	2 (6.5%)	5 (17.2%)	8 (25%)	4 (23.5%)	19 (17.4%)
T15	Witnessing violence	8 (25.8%)	9 (31.0%)	22 (68.8%)	14(82.4%)	53 (48.6%)
T16	Family mental illness	8 (25.8%)	10 (34.5%)	17 (53.1%)	7 (41.2%)	42 (38.5%)
T17	Alcoholic parents	3 (9.7%)	4 (13.8%)	11 (34.4%)	7 (41.2%)	25 (22.9%)
T18	Drug abuse in parents	2 (6.5%)	1 (3.5%)	2 (6.3%)	0 (0%)	5 (4.6%)
T19	Victim of major theft	12 (38.7%)	13 (44.8%)	17 (53.1%)	9 (52.9%)	51 (46.8%)
T20	Victim of armed robbery	3 (9.7%)	0 (0%)	7 (21.9%)	6 (35.3%)	16 (14.7%)
T21	Victim of assault	1 (3.2%)	4 (13.8%)	13 (40.6%)	5 (29.4%)	23 (21.1%)
T22	Victim of rape	1 (3.2%)	0 (0%)	4 (12.5%)	3 (17.7%)	8 (7.3%)
T23	See someone murdered	1 (3.2%)	0 (0%)	2 (6.3%)	2 (11.8%)	5 (4.6%)
T24	Someone close to you murdered	1 (3.2%)	1 (3.5%)	3 (9.4%)	4 (23.5%)	9 (8.3%)
T25	Someone close to you raped	2 (6.5%)	1 (3.5%)	4 (12.5%)	4 (23.5%)	11 (10.1%)
T26	Work in stressful job	1 (3.2%)	4 (13.8%)	9 (28.1%)	5 (29.4%)	19 (17.4%)
T27	POW/hostage	0 (0%)	0 (0%)	0 (0%)	2 (11.8%)	2 (1.8%)
T28	Combat	0 (0%)	1 (3.5%)	1 (3.1%)	2 (11.8%)	4 (3.7%)
T29	Death of child	0 (0%)	1 (3.5%)	2 (6.3%)	0 (0%)	3 (2.8%)
T30	Miscarriage	0 (0%)	0 (0%)	4 (12.5%)	1 (5.9%)	5 (4.6%)
T31	Death of spouse	-	-	-	-	-

### Emotional abuse

The most prevalent childhood emotional abuse experienced in the sample was being often shouted or yelled at (*n* = 58, 53.2%), followed by being often put down or ridiculed (*n* = 55, 50.5%), and parental failure to understand the participant's needs as a child (*n* = 53, 48.6%). The most prevalent trauma in the SAD with moderate/severe EDT group, (*n* = 26, 81.3%)was a lack of understanding of the participant's needs and being often put down or ridiculed bya parent (*n* = 26). This was followed by often being ignored or made to feel that the person did not count (*n* = 24, 75%), and often being shouted or yelled at (*n* = 22, 68.8%). Within the PTSD group, the most prevalent form of emotional abuse experienced as a child was often being shouted or yelled at (*n* = 12, 70.6%), followed by being put down or ridiculed (*n* = 11, 64.7%), and often ignored or made to feel like the person did not count (*n* = 11, 64.7%). The most prevalent emotional abuse experienced in the SAD with low/no EDT group and control groups was being often shouted or yelled at 29% (*n* = 9) and 51.7% (*n* = 15), respectively (see Table 3).

### Sexual abuse

The most prevalent form of sexual abuse experienced was being touched in intimate parts in a way that was uncomfortable (*n* = 30, 27.5%), followed by being exposed to inappropriate comments about sex (*n* = 28, 25.7%), and being exposed to flashing (*n* = 22, 20.2). The most prevalent childhood sexual abuse experienced by the SAD with moderate/severe EDT group was exposure to inappropriate comments about sex (*n* = 13, 40.6%), followed by exposure to flashing (*n* = 12, 37.5%), and being touched in intimate parts in a way that was uncomfortable (*n* = 12, 37.5%). The most prevalent sexual abuse experienced by the PTSD group was being touched in intimate parts in a way that was uncomfortable (*n* = 10, 58.8%), followed by exposure to inappropriate comments about sex (*n* = 47.1%), and someone trying to have sex with the individual, but not doing so (*n* = 7, 41.2%). In the SAD group with low/no EDT, the most prevalent childhood sexual abuse experienced was being exposed to inappropriate comments about sex (*n* = 4, 13.8%). The most prevalent sexual abuse experienced by the control group was being touched in intimate parts in a way that was uncomfortable (*n* = 6, 19.35%) (see Table 3).

### Other childhood traumatic events (defined as general trauma on the ETI)

In the sample as a whole, the most prevalent ‘other’ childhood traumatic life event was witnessing violence (*n* = 52, 48.6%), followed by being a victim of major theft (*n* = 51, 46.5%). This was followed by family mental illness (*n* = 42, 38.5%) and serious injury/illness of a parent (*n* = 41, 37.6%). Witnessing violence was the most prevalent traumatic life event in both the SAD with moderate/severe EDT (*n* = 22, 68.7%) and the PTSD (*n* = 14, 82.35%) groups. In the SAD with low/no EDT group, the most prevalent traumatic childhood event was being a victim of major theft (*n* = 13, 44.85), followed by family mental illness (*n* = 10, 34.5%), and then witnessing violence (*n* = 9, 31%). In the control group, the most prevalent traumatic childhood event was being a victim of major theft (*n* = 12, 38.7%) and experiencing a serious personal injury (*n* = 11, 35.5%) (see Table 3).

### Age of onset

In all groups the age of onset of physical and emotional abuse was generally between the age of 6 and 11 years. Sexual abuse in the SAD with moderate/severe EDT and control groups tended to occur between 13 and 18 years of age, while in the PTSD group the age of onset was earlier (6–11 years) (Table 4).

**Table 4:** Age of onset of childhood traumatic events as measured by the ETI

	**Controls**				**SAD with EDT**				**PTSD**			
	**Never**	**0–5**^**a**^	**6–11**^**b**^	**13–18**^**c**^	**Never**	**0–5**^**a**^	**6–11**^**b**^	**13–18**^**c**^	**Never**	**0–5**^**a**^	**6–11**^**b**^	**13–18**^**c**^
Physical abuse events												
Spanked with hand	5	11	14	1	2	14	14	2	2	8	6	1
Slapped in face	23	2	3	3	9	1	11	11	9	1	3	4
Burned with cigarette	31	0	0	0	29	2	1	0	13	2	2	0
Punched or kicked	24	0	1	6	18	1	9	4	11	1	2	3
Hit or spanked with object	13	4	9	5	7	10	12	3	1	4	11	1
Hit with thrown object	28	0	1	2	22	1	5	4	11	0	5	1
Choked	31	0	0	0	25	1	2	4	14	1	0	2
Pushed or shoved	22	0	4	5	17	1	7	7	8	0	5	4
Tied up or locked in closet	30	1	0	0	29	1	2	0	15	1	1	0
Total scores	207	18	32	22	158	32	63	35	84	18	35	16
Emotional abuse events												
Often put down or ridiculed	24	0	2	5	6	5	14	7	6	2	5	4
Often ignored or made to feel you didn't count	27	0	3	1	8	8	9	7	6	5	6	0
Often told you are no good	26	0	3	2	16	0	9	7	8	2	4	3
Often shouted or yelled at	22	2	5	2	9	8	11	4	5	4	6	2
Most of the time treated in cold or uncaring way	29	1	0	1	13	2	12	5	8	3	5	1
Parents control areas of your life	22	2	6	1	13	5	10	4	8	2	6	1
Parents fail to understand your needs	27	1	1	2	6	6	12	8	7	2	5	3
Total scores	177	6	20	14	71	34	77	42	48	20	37	14
Sexual abuse events												
Exposed to inappropriate comments about sex	28	0	3	0	19	3	5	5	9	0	4	4
Exposed to flashing	29	1	0	1	20	4	4	4	11	0	3	3
Spy on you dressing/bathroom	28	1	0	2	24	2	4	2	15	0	2	0
Forced to watch sexual acts	31	0	0	0	27	0	3	2	16	0	1	0
Touched in intimate parts in way that was uncomfortable	25	0	3	3	20	2	5	5	7	1	4	5
Someone rubbing genitals against you	29	0	1	1	24	2	3	3	12	0	3	2
Forced to touch intimate parts	30	0	0	1	28	1	1	1	12	0	3	2
Someone had genital sex against your will	30	0	0	1	29	1	2	0	13	1	1	2
Forced to perform oral sex	31	0	0	0	31	0	1	0	15	0	2	2
Someone performed oral sex on you against your will	31	0	0	0	29	0	1	1	15	0	2	0
Someone had anal sex with you against your will	31	0	0	0	30	1	0	1	15	0	1	1
Someone tried to have sex but didn't do so	30	0	0	1	23	1	3	4	10	1	3	3
Forced to pose for sexy photographs	31	0	0	0	31	0	0	1	15	0	1	1
Forced to perform sex acts for money	31	0	0	0	0	0	0	0	0	0	0	0
Forced to kiss someone in sexual way	31	0	0	0	23	1	2	6	15	0	0	2
Total scores	446	2	7	10	358	18	34	35	180	3	30	27

^a^Between and including the ages 0–5 years; ^b^between and including the ages 6–11 years; ^c^between and including the ages 13–18 years.

### Perpetrators

For physical and emotional abuse, in all groups, the perpetrator was mostly the primary caregiver. In both the SAD with moderate/severe EDT and control groups this was a female caregiver, while for the PTSD group a male primary caregiver was mostly reported. For emotional abuse, the perpetrator most commonly reported by all groups was a female primary caregiver. With regards to the perpetrator of sexual abuse, the most frequently reported perpetrator by all groups was another male adult (Tables 5 and 6).

**Table 5:** Relationship to perpetrator as measured by the ETI

	**Controls**				**SAD with EDT**				**PTSD**			
	**Caregiver**	**Other adult**	**Sibling**	**Stranger**	**Caregiver**	**Other adult**	**Sibling**	**Stranger**	**Caregiver**	**Other adult**	**Sibling**	**Stranger**
Physical abuse events												
Spanked with hand	25	1	0	0	28	1	1	0	14	1	0	0
Slapped in face	8	0	0	0	15	6	1	2	6	2	0	0
Burned with cigarette	0	0	0	0	3	0	0	0	1	1	0	2
Punched or kicked	2	0	2	3	4	1	3	6	2	3	1	0
Hit or spanked with object	12	4	1	1	23	0	1	1	16	0	0	0
Hit with thrown object	1	1	1	0	7	0	2	1	3	0	2	1
Choked	0	0	0	0	1	1	2	3	2	1	0	0
Pushed or shoved	1	1	2	5	5	3	2	5	2	1	4	2
Tied up or locked in closet	0	1	0	0	2	1	0	0	2	0	0	0
Total scores	49	8	6	9	88	13	12	18	48	9	7	5
Emotional abuse events												
Often put down or ridiculed	3	1	2	1	16	3	4	4	8	1	1	1
Often ignored or made to feel you didn't count	1	0	3	0	14	6	3	1	9	2	0	0
Often told you are no good	2	1	2	0	11	2	1	2	7	0	2	0
Often shouted or yelled at	7	1	1	0	20	1	1	1	11	5	4	1
Most of the time treated in cold or uncaring way	0	1	1	0	16	1	2	0	8	1	0	0
Parents control areas of your life	9	0	0	0	18	1	0	0	8	1	0	0
Parents fail to understand your needs	4	0	0	0	25	1	0	0	9	1	0	0
Total scores	26	4	9	1	120	15	11	8	60	11	7	2
Sexual abuse events												
Exposed to inappropriate comments about sex	0	3	0	0	5	7	0	1	0	7	1	0
Exposed to flashing	1	0	0	1	1	7	0	5	0	4	1	1
Spy on you dressing/bathroom	2	0	0	1	3	3	1	1	0	2	0	0
Forced to watch sexual acts	0	0	0	0	1	3	0	2	0	1	0	0
Touched in intimate parts in way that was uncomfortable	1	4	0	1	1	5	0	6	0	8	1	1
Someone rubbing genitals against you	1	0	0	0	1	4	0	3	0	4	0	1
Forced to touch intimate parts	0	1	0	0	0	2	0	1	0	5	0	0
Someone had genital sex against your will	0	1	0	0	0	1	0	2	0	4	0	0
Forced to perform oral sex	0	0	0	0	0	1	0	0	0	2	0	0
Someone performed oral sex on you against your will	0	0	0	0	0	2	0	0	0	1	0	1
Someone had anal sex with you against your will	0	0	0	0	0	1	0	1	0	1	0	1
Someone tried to have sex but didn't do so	0	1	0	0	1	4	0	3	0	7	0	0
Forced to pose for sexy photographs	0	0	0	0	0	1	0	0	0	2	0	0
Forced to perform sex acts for money	0	0	0	0	0	0	0	0	0	0	0	0
Forced to kiss someone in sexual way	0	0	0	0	2	5	0	2	0	2	0	0
Total scores	5	10	0	3	15	46	1	27	0	50	3	5

**Table 6:** Gender of perpetrators (primary caregivers and other adults)

	**Controls**				**SAD with EDT**				**PTSD**			
	**Primary caregivers**		**Other adults**		**Primary caregivers**		**Other adults**		**Primary caregivers**		**Other adults**	
	**F**	**M**	**F**	**M**	**F**	**M**	**F**	**M**	**F**	**M**	**F**	**M**
Physical abuse events												
Spanked with hand	16	9	1	0	17	11	0	1	7	7	0	1
Slapped in face	6	2	0	0	9	6	2	4	1	5	0	2
Burned with cigarette	0	0	0	0	0	3	0	0	0	1	0	1
Punched or kicked	0	2	0	0	1	3	0	1	0	2	0	3
Hit or spanked with object	8	4	3	1	13	10	0	0	12	4	0	0
Hit with thrown object	1	0	0	1	4	3	0	0	2	1	0	0
Choked	0	0	0	0	1	0	0	1	0	2	0	1
Pushed or shoved	0	1	0	1	2	3	1	2	0	2	0	1
Tied up or locked in closet	0	0	0	1	1	1	0	1	0	1	0	0
Total scores	31	18	4	4	49	40	3	10	22	25	0	9
Emotional abuse events												
Often put down or ridiculed	1	2	1	0	9	7	1	2	4	4	0	1
Often ignored or made to feel you didn't count	1	0	0	0	7	7	3	3	5	4	1	1
Often told you are no good	2	0	1	0	4	7	0	2	3	4	0	0
Often shouted or yelled at	4	3	1	0	15	5	0	1	7	4	1	0
Most of the time treated in cold or uncaring way	0	0	0	1	11	5	0	1	4	4	1	0
Parents control areas of your life	8	1	0	0	17	1	0	1	6	2	1	0
Parents fail to understand your needs	3	1	0	0	18	7	0	1	5	4	1	0
Total scores	19	7	3	1	81	39	4	11	34	26	5	2
Sexual abuse events												
Exposed to inappropriate comments about sex	0	0	0	3	1	4	2	5	0	0	2	5
Exposed to flashing	1	0	0	0	0	1	1	6	0	0	1	3
Spy on you dressing/bathroom	2	0	0	0	2	1	0	3	0	0	0	2
Forced to watch sexual acts	0	0	0	0	0	1	0	3	0	0	1	0
Touched in intimate parts in way that was uncomfortable	0	1	0	4	0	1	0	5	0	0	1	7
Someone rubbing genitals against you	0	1	0	0	0	1	0	4	0	0	1	3
Forced to touch intimate parts	0	0	0	1	0	0	0	2	0	0	1	4
Someone had genital sex against your will	0	0	0	1	0	0	0	1	0	0	1	3
Forced to perform oral sex	0	0	0	0	0	0	0	1	0	0	1	1
Someone performed oral sex on you against your will	0	0	0	0	0	0	0	2	0	0	1	0
Someone had anal sex with you against your will	0	0	0	0	0	0	0	1	0	0	0	1
Someone tried to have sex but didn't do so	0	0	0	1	0	1	0	4	0	0	1	6
Forced to pose for sexy photographs	0	0	0	0	0	0	0	1	0	0	1	1
Forced to perform sex acts for money	0	0	0	0	0	0	0	0	0	0	0	0
Forced to kiss someone in sexual way	0	0	0	0	1	1	0	5	0	0	1	1
Total scores	3	2	0	10	4	11	3	43	0	0	13	37

*F* female, *M* male.

### Correlation between ETI and CTQ

Significant positive correlations were found between all childhood traumas measured on the CTQ (physical abuse, physical neglect, emotional abuse, emotional neglect, and sexual abuse) and the ETI (physical abuse, general abuse, sexual abuse, and emotional abuse) (see Table 7). Nine high correlations (identified as correlation coefficients *r* > 0.70) were found and are reported in descending order from the strongest correlations to a correlation coefficient of 0.70 [34]. These were (i) childhood emotional abuse (CTQ EA) and total trauma (CTQ total score) (*r* = 0.87, *p* = 0.00), (ii) childhood emotional neglect (CTQ EN) and total trauma (CTQ total score) (*r* = 0.85, *p* = 0.00), (iii) other traumas experienced during childhood (ETI general trauma score) and total trauma (ETI total score) (*r* = 0.85, *p* = 0.00), (iv) emotional abuse during childhood (ETI EA score) and total childhood trauma (ETI total score) (*r* = 0.76, *p* = 0.00), (v) childhood physical abuse (CTQ PA) and total childhood trauma (CTQ total score) (*r* = 0.74, *p* = 0.00), (vi) childhood physical neglect (CTQ PN score) and total trauma (CTQ total score) (*r* = 0.73, *p* = 0.00), (vii) childhood total trauma (CTQ total score) and total trauma on the ETI total score (*r* = 0.72, *p* = 0.00), (viii) childhood sexual abuse (CTQ SA) and childhood sexual abuse measured by the ETI (*r* = 0.71, *p* = 0.00), and (ix) childhood emotional abuse on the ETI and total trauma on the CTQ (*r* = 0.70, *p* = 0.00) (see Table 7).

**Table 7:** Spearman's nonparametric correlations between childhood traumas measured by CTQ and ETI

**Variable**	**Spearman's rho (*r *** **value) and *p *** **values**	**Physical abuse (CTQ)**	**Sexual abuse (CTQ)**	**Emotional neglect (CTQ)**	**Physical neglect (CTQ)**	**Total childhood trauma (CTQ)**	**General trauma (ETI)**	**Physical abuse (ETI)**	**Emotional abuse (ETI)**	**Sexual abuse (ETI)**	**Total childhood trauma (ETI total)**
Emotional abuse (CTQ)	*r* value	0.65	0.47	0.66	0.54	0.87	0.48	0.47	0.69	0.46	0.68
	*p* value	0.00**	0.00**	0.00**	0.00**	0.00**	0.00**	0.00**	0.00**	0.00**	0.00
Physical abuse (CTQ)	*r* value		0.37	0.52	0.47	0.74	0.35	0.56	0.45	0.33	0.53
	*p* value		0.00**	0.00**	0.00**	0.00**	0.00**	0.00**	0.00**	0.00**	0.00**
Sexual abuse (CTQ)	*r* value			0.38	0.44	0.60	0.41	0.28	0.26	0.71	0.51
	*p* value			0.00**	0.00**	0.00**	0.00**	0.00**	0.01**	0.00**	0.00**
Emotional neglect (CTQ)	*r* value				0.58	0.85	0.27	0.37	0.63	0.37	0.51
	*p* value				0.00**	0.00**	0.01**	0.00**	0.00**	0.00**	0.00**
Physical neglect (CTQ)	*r* value					0.73	0.43	0.29	0.42	0.39	0.49
	*p* value					0.00**	0.00**	0.00**	0.00**	0.00**	0.00**
Total childhood trauma (CTQ)	*r* value						0.49	0.51	0.70	0.54	0.72
	*p* value						0.00**	0.00**	0.00**	0.00**	0.00**
General trauma (ETI)	*r* value							0.45	0.48	0.49	0.85
	*p* value							0.00**	0.00**	0.00**	0.00**
Physical abuse (ETI)	*r* value								0.47	0.27	0.69
	*p* value								0.00**	0.00**	0.00**
Emotional abuse (ETI)	*r* value									0.36	0.76
	*p* value									0.00**	0.00**
Sexual abuse (ETI)	*r* value										0.63
	*p* value										0.00**

**Correlations are significant at the 0.01 level (two-tailed). CTQ, *n* = 108; ETI, *N* = 109.

## Discussion and conclusions

The main objective of this study was to investigate differences in the type and amount of childhood trauma in adults with PTSD and SAD. No statistically significant differences were found for these disorders.

This finding suggests that in this sample, childhood traumas (major ALE, physical, sexual, emotional, and total) are not significantly different in individuals with PTSD and SAD with moderate/severe EDT. Previous studies have found that physical abuse [10,12,13], sexual abuse [12-18], emotional abuse [19-23], as well as other childhood adversities [24,25] are linked to the development of both PTSD and SAD. Childhood emotional abuse has been found to correlate more strongly with a diagnosis of social anxiety than either physical or sexual abuse [20]. Previous studies have also documented higher rates of childhood emotional abuse and neglect in adults with SAD compared to healthy controls [21,22]. Emotional abuse and neglect significantly impact on the development of SAD and PTSD [23]. The results of our study regarding emotional abuse indicates that parents' emotional expression toward their children can have long-lasting effects and contribute to PTSD and SAD in later life. The aforementioned studies, however, did not specifically compare EDT exposure, by severity and type, in these disorders.

### Age of onset

With regards to the onset of childhood abuse, Rodriguez et al. in 1996 also found the age of onset of sexual abuse in individuals with PTSD to be around 6 years of age and termination at approximately 13 years, which is similar to our finding in the PTSD group of between 6 and 11 years [27]. This finding is supported by two other studies that found the age of onset of childhood sexual abuse to be approximately 7 years [26] and 9.85 years [28], respectively. There is a paucity of studies on the age of onset of EDT in people with PTSD and SAD, underscoring the need for more investigation in this area to better inform the nature and timing of interventions.

### Perpetrators

With regards to the perpetrators, our finding of sexual abuse mostly being another nonfamilial male adult is supported by the findings in a previous study of perpetrators of EDT [27]. According to Rodriguez et al. in 1996, perpetrators of CSA in individuals with PTSD were mostly male (87%), nuclear family members, followed by nonfamily members, and lastly by extended family members [27]. Also, Feerick and Snow in 2005 showed in their study that participants were mostly sexually abused by other perpetrators, such as acquaintances, boyfriends, and babysitters, i.e., nonfamilial persons and strangers [28]. Ackerman et al. in 1998 found that if a child was physically abused by a male, the likelihood of a psychiatric diagnosis, including PTSD, was higher than if abused by a female [26]. Overall, physically and sexually abused children were more likely to develop a psychiatric disorder which included PTSD in later life. No further studies focusing on perpetrators of EDT in individuals with SAD or PTSD were found, highlighting a gap that warrants attention in future studies.

### ETI and CTQ

All traumas measured by the CTQ were positively correlated with all traumas measured by the ETI. Kuo et al. in 2011 also found that all scales on the CTQ had a significant positive correlation with all subscales, except for sexual abuse and emotional abuse or neglect [21]. Bremner et al. in 2000 tested the convergent validity of the ETI by comparing its domains (physical, sexual, and general) with the components of the Childhood Trauma Severity Index (CLTE) [33]. Significant correlations were calculated between the total score of the ETI and the total score of the CLTE, the physical abuse domain of the ETI and the physical abuse component of the CLTE, as well as between the sexual abuse domain of the ETI and the sexual abuse component of the CLTE [33].

### Limitations and recommendations

A number of limitations warrant mention. Firstly, the results of the LSD *post hoc* testing should be interpreted with caution given that this type of analysis is associated with type 1 error. Secondly, group sizes were small. Thirdly, endorsement of EDT was reliant on the retrospective accounts of participants. This may not always be very accurate with regards to memory recall of traumatic early life events. Recall bias is, therefore, a concern. Recall may also have been affected by the way in which childhood events were assessed [25]. It has been suggested that rather than an exaggeration in the rates, childhood traumas are more likely to be under-reported [35]. In addition, participants varied in age and older participants in particular may have had more difficulty recalling childhood events [36]. The sample may not be demographically representative of the South African population with SAD and PTSD with early trauma. Future studies should include more Black African participants. Furthermore, studies of larger samples that include more qualitative assessments of childhood trauma are needed. In sum, the contribution of EDT to the development of PTSD and SAD and the differences in terms of childhood trauma between these groups, as well as other anxiety disorders, should not be ignored, and attention should be given to the frequency and severity of these events. The relationship between victims of EDT and perpetrators and the age of onset of childhood abuse is another important facet, as it provides a timeline against which the course of abuse and its impact can be tracked over the life trajectory. This can, in turn, provide some guidance to clinicians on the optimal timing and nature of interventions for the prevention of PTSD and SAD.

## Competing interests

The authors declare that they have no competing interests.

## Authors' contributions

MB participated in drafting and revising the manuscript, data analysis, interpretation of data, made a substantial contribution to the acquisition of data, and ensured that questions related to the accuracy of the work are appropriately resolved. DR participated in the design of the study, performed statistical analysis, critically revising the manuscript, and made a substantial contribution on the acquisition of data (all clinical data). SB participated in the design of the study, data acquisition, and critically revising the manuscript. SS conceptualized the study and made substantial contributions to the conception and design, analysis of data, revised the manuscript, and provided the final approval of the version to be published. All authors read and approved the final manuscript.
